# Postharvest Practices and Farmers’ Knowledge in Managing Maize Pests in the Eastern Cape Province, South Africa

**DOI:** 10.3390/insects16010048

**Published:** 2025-01-06

**Authors:** Bongumusa Charles Gumede, Simon Kamande Kuria

**Affiliations:** Department of Biological and Environmental Sciences, Faculty of Natural Sciences, Walter Sisulu University, Mthatha 5117, South Africa; 216250897@mywsu.ac.za

**Keywords:** postharvest, *Sitophilus zeamais*, aluminium phosphide, smallholder farmers

## Abstract

This study aims to establish the pest management approach for pests of stored maize and determine the current control practices. Semi-structured questionnaires were administered to 77 smallholder farmers from 16 villages at King Sabata Dalindyebo local municipality in the Eastern Cape Province of South Africa. The results revealed that about 50% of the farmers had a formal education, the average farm size was 1 hectare, and they were predominantly cultivating yellow maize. Most farmers (57%) cultivated maize for human consumption, income generation, and livestock feeding. The results also showed that metal tanks tended to be the most preferred (81.82%) storage facility for storing maize. Furthermore, the study found that *Sitophilus zeamais* Motschulsky and *Sitotroga cerealella* (Olivier) were the major storage insect pests of maize. The storage pests were managed using synthetic pesticides (84.42%), namely aluminium phosphide. This pesticide is a solid formulation for generating gas phosphine for fumigation. The continued indiscriminate use of this pesticide in protecting stored maize in this region could be a health hazard to humans and may result in the development of pest resistance.

## Simple Summary

The current study intends to establish the pest management approach for smallholder maize farmers on storage pests of maize and determine their current control practices. We administered a questionnaire to maize farmers from the Eastern Cape Province of South Africa. The study found that metal tanks are the farmers most preferred storage facility of maize. The results showed that maize was mostly infested by maize weevils and maize grain moths. Farmers reported managing these pests using chemical pesticides. The extensive usage of this pesticide in protecting stored maize could cause human health-related issues and may result in the development of pest resistance.

## 1. Introduction

Maize cultivation performs well in a vast range of regions, including the tropics, subtropics, and temperate zones [1]. Albeit maize is predominantly grown in Latin America, Asia and sub-Saharan African countries like South Africa also grow it, where it plays a pivotal role in food security and the agricultural economy [2]. The total amount of maize production worldwide is approximately 1.137 million metric tonnes [2]. In South Africa, maize is the most important staple crop in terms of production and consumption [3]. Farmers normally rely on their storage techniques and knowledge to preserve maize during the long off-season period before the next harvest [4].

Maize storage practices in South Africa among commercial and smallholder farmers vary significantly and are influenced by economic resources and access technologies [5]. Commercial maize farmers predominantly use metal silos for grain storage, which is considered the most effective method in South Africa in protecting maize [6]. On the other hand, smallholder farmers mostly use traditional silos, metal containers, and sacks [7]. Nonetheless, these maize storage methods used by smallholder farmers are ineffective except for metal containers [8].

Maize plays a major role in the food security of small-scale farmers as a food and cash crop for millions of rural farm families [9]. However, this beneficial food material has several challenges [10]. Studies in sub-Saharan Africa, which includes South Africa, have shown that maize is susceptible to infestation by rodents, fungi, and insect pests during the storage period [11]. For example, farmers and workers postulated that a rodent infestation is influenced by the presence of waste and spilled grains near grain storage facilities [12]. Further, research findings have elucidated that poor postharvest practices and storage facilities have extensively contributed to maize grain being contaminated by mycotoxin due to fungal infestations. Similarly, high temperatures and poor storage conditions favour insect pest infestations [13].

Some of the major primary insect pests of maize include maize weevils, *Sitophilus zeamais* Motschulsky (Coleoptera: Curculionidae), rice weevils, *Sitophilus oryzae* (Linnaeus) (Coleoptera: Curculionidae), maize grain moths, *Sitotroga cerealella* (Olivier) (Lepidoptera: Gelechiidae), and larger grain borers *Prostephanus truncatus* (Horn) (Coleoptera: Bostrichidae) that directly contribute significantly to postharvest losses through feeding and reproduction, impacting the quality and quantity of stored grain [14]. Indirectly, the presence and feeding of these insect pests can elevate the grain temperature and moisture levels stimulating grain deterioration and enhancing fungal activity [15].

Consequently, primary insect pests attract secondary insect pests of the maize such as the rust-red flour beetle, *Tribolium castaneum* (Herbst) (Coleoptera: Terebrionidae), flat grain beetle, *Cryptolestes pusillus* (Schonherr) (Coleoptera: Cucujidae), saw-toothed grain beetle, *Oryzaephilus surinamensis* (Linnaeus) (Coleoptera: Silvanidae), and grain mites, *Acarus siro* (Linnaeus) (Acarina: Acaridae) which cannot damage the whole grain but infest grains that are either mechanically damaged or previously infested by primary pests [16].

Various methods have been used in the management of these insect pests, including chemical control, biological control, cultural control, and others [17]. Nonetheless, the use of synthetic pesticides like aluminium phosphide, malathion, ethyl formate, etc., has been reported as the commonly preferred method in managing the insect pests of maize [18]. However, the extensive usage of these pesticides results in a resistance to targeted pests like maize weevils, reducing their effectiveness [19]. Moreover, there are growing concerns on human health hazards and the contamination of the environment at large due to the excessive use of inorganic pesticides [20]. Thus, there is a need to develop an integrated pest management approach that is safe and cost-effective for smallholder farmers [21].

In spite of these challenges caused by storage pests of maize, there is a paucity of information on farmers’ knowledge and practices in managing these pests in some parts of the Eastern Cape province [22]. To establish effective pest management approaches for smallholder farmers, this study aims to delineate their knowledge and current control practices. Thus, the objectives of the current study were to, as follows: (1) document methods used by smallholder farmers of storing maize; (2) evaluate farmers’ knowledge of the storage pests of maize; and (3) determine practices used by farmers in managing storage pests of maize.

## 2. Materials and Methods

### 2.1. Study Area

The study was conducted in King Sabata Dalindyebo local municipality (KSDM) (31°38′15.8″ S, 28°30′30.1″ E) under Oliver Reginald Tambo District Municipality (ORTDM) in the Province of Eastern Cape, South Africa. The specific study sites in KSDM were 16 villages (Baziya, Nyibeni, Ndibela, Ngcotyeni, Bityi, Gunjane, Mokolweni, Etafeni, Kukhambi, Kugutya, Manyosini, Chanti, Qunu, Krakra, Noncwenga, and Mayenge). According to the KSDM annual report [23], the KSDM is the largest from the five local municipalities within ORTDM in terms of surface area, which covers about 302,700 hectares with an approximately 520,000 human population. The annual rainfall in the ORTDM varies, but on average, it is about 900 millimetres per year [24]. Rainfall is usually higher during the summer months, between December and February, while winters are generally drier. Farming systems are diverse with subsistence farming being common among local communities. Additionally, maize, sorghum, beans, and vegetables are among the staple crops grown by smallholder farmers [25].

### 2.2. Data Collection

The questionnaire survey was conducted from November 2023 to March 2024 to determine farmers’ current knowledge and control practices for maize storage insect pests. Purposive sampling was conducted, where farmers who are willing to participate were selected from 16 villages. The list of the farmers came from the Ukhanyo Farmer Development (UFD) and the Department of Rural Development and Agrarian Reform (DRDAR). A semi-structured questionnaire with both open- and closed-ended questions were administered to 77 smallholder farmers. Prior administering the questionnaire, the purpose of the study was explained to all of the farmers and written consent was obtained. The questionnaire was piloted using 10 farmers to ensure its validity. The information sought includes the following: (i) farmers socio-demographic profile, (ii) farm characteristics, (iii) purpose of farming maize, and (iv) facilities used to store maize and their knowledge on stored maize pests, and (v) control practices used for maize weevils.

### 2.3. Data Analysis

Questionnaire data were summarized and a descriptive data analysis was conducted using frequencies and percentages in Microsoft excel 365.

## 3. Results

### 3.1. Socio-Demographic Characteristics of Farmers

A total of 77 farmers from 16 villages in ORTDM in the Eastern Cape Province (ECP) participated in the current study. The results show that the majority of the maize farmers were males (71.43%) and most of the farmers (62.34%) were elderly people above 56 years (Table 1). Over 80% of the farmers had received a formal education and 76.62% had 10 years of farming experience (Table 1).

### 3.2. Farm Characteristics

**Figure 1:**
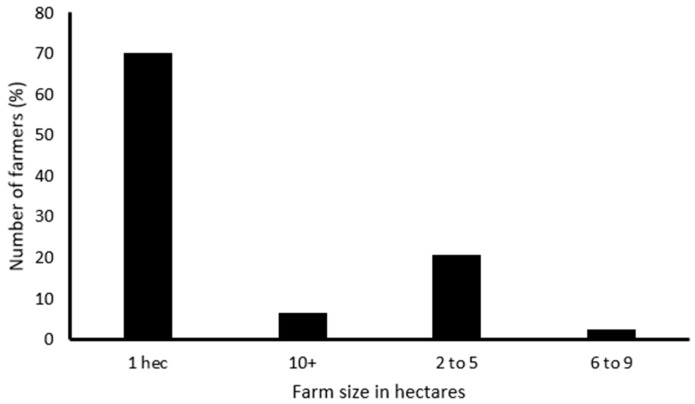
Farm size under maize cultivation in KSD municipality.

**Figure 2:**
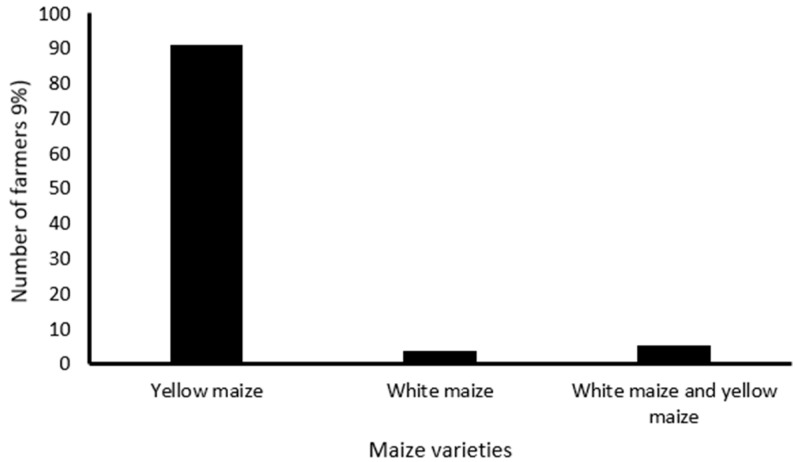
Maize varieties cultivated in KSD municipality.

The results shows that most farmers (70.13%) had 1 hectare (Figure 1) and predominantly used their land for cultivating yellow maize (90.91%) (Figure 2). 

### 3.3. Purpose of Farming

**Figure 3:**
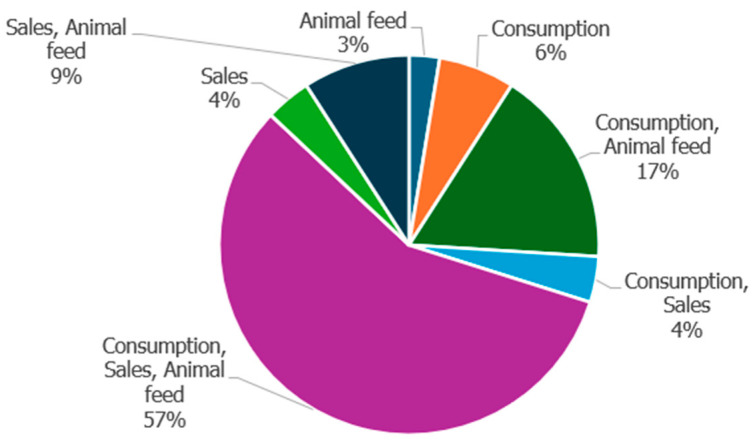
Percentage of farmers and how they use stored maize.

Maize was mainly grown and stored for human consumption, income generating, and for feeding livestock by most farmers (57%) (Figure 3), whereas 3% of the farmers used their maize as livestock feed (Figure 3).

### 3.4. Facilities Used to Store Maize, Storage Forms of Maize, Farmers’ Knowledge of Storage Pests, and Control Practices

A large proportion of farmers (81.82%) store their maize in metal tanks and about 97.40% of farmers store their maize in shelled form, which they reported is mostly infested by maize weevils (89.61%) and maize grain moths (74.03%) (Table 2). In this region, farmers managed maize storage pests mostly through phosphine fumigation in the form of aluminium phosphide tablets (84.42%) (Table 2).

## 4. Discussion

The current study shows that more males were participating in maize farming than females. This result agrees with other research findings on gender differences in agriculture. For example, Agbugba et al. [26] reported that in the Amathole District in ECP from a total of 109 farmers, 66.1% were males. Similarly, Kibirige [27] indicated that in the Chris Han District in ECP, 66% of farmers were males. These findings can be attributed to the fact that most households are headed by men [24]. Additionally, agricultural land is normally allocated more to males than females due to sociocultural norms and patriarchal constructs [28].

Regarding age, education, and farming experience, the current study found that the majority of farmers were elderly people above 56 years and most of them had a formal education (primary, secondary, and tertiary), with more than 10 years of farming experience. Similar findings were reported by Afolayan et al. [29], who found that most farmers in the OR Tambo District, Amathole District, and Chris Han District in ECP were above 56 years and more than 90% of farmers had a formal education with over 10 years of farming experience. These results may be true in the fact that farming is practiced by an older age group of people in the Eastern Cape Province because the youth in rural areas are migrating to cities in search of better living and opportunities [30]. On the other hand, education and farming experience play a pivotal role for farmers in understanding and adopting sustainable practices for effective farming performance [31].

Farm size is another factor that may affect the productivity and growth of farmers, as in the current study, most farmers own small plots of land for farming with a farm size of 1 hectare, and primarily use their land for cultivating mostly yellow maize. Furthermore, most farmers produce and store yellow maize for human consumption, as a source of income, and for feeding livestock. These results are in consonance with those reported by Mdoda and Gidi [32], which demonstrate that the majority (60%) of smallholder farmers from the OR Tambo, Amathole, and Chris Hani Districts in ECP have their farm size ranging from 0.5 to 2.5 hectares and 50.6% of them are growing maize. Moreover, Sibanda et al. [33] reported that the majority (70.8%) of smallholder farmers preferred yellow maize to white maize. This can be attributed to the fact that yellow maize is multipurpose by feeding livestock, poultry, and being for human consumption, which makes it have a better market value [33,34]. This shows that maize has a multifunctional role in agriculture and significantly contributes to household food security [35].

The current study found that many farmers store their maize in metal tanks in a shelled form. Conversely, Thamaga-Chitja [8] reported about 20 years ago that 52% of farmers in KwaZulu Natal used traditional silos to store maize as opposed to metal tanks and sacks. The results show that the farmers are progressively moving from the use of traditional storage techniques to modern ones [23]. The reason being for this is that the modern techniques for storing maize, such as hermetic bags and metal containers, offer noteworthy advantages in reducing postharvest losses, particularly from insect pests [36]. However, they present challenges such as the high initial cost and advanced knowledge for implementation, as that can impede their adoption by smallholder farmers [18]. Furthermore, Mendoza et al. [37], also reported that 90% of farmers dry their maize and store them in a shelled form because shelled maize is less prone to spoilage and infestation by pests. The removal of the protective husk lowers the moistness and provides less conducive conditions for microbial growth [38].

The present study found that stored maize in this region (ECP) is mostly infested by maize weevils (*S. zeamais*), followed by maize grain moths (*S. cerealella*). Subsequently, farmers protect their maize from storage pests mostly through fumigation in the form of aluminium phosphide tablets. Similarly, Megerssa et al. [39], reported that in Ethiopia, about 58.7% of farmers had insects as their most common pest of stored maize, with maize weevils being the most important followed by maize grain moths. Furthermore, Megerssa et al. [39] observed most farmers (75.3%) use inorganic pesticides like aluminium phosphide compared to traditional practices (17%) like the use of botanical biopesticides and cultural practices. Their findings support those reported in this study.

Despite the effective contribution of aluminium phosphide in pest management, its indiscriminate usage has led to acute and chronic harmful effects on humans and the development of resistance by insect pests [40]. The side effects are likely to affect maize farmers in the Eastern Cape, South Africa. To safeguard aluminium phosphide poisoning, farmers should implement safety measures like participating in training programs and using personal protective equipment (PPE) when using this chemical [41,42]. We intend to start sensitizing maize farmers in this region and propose training workshops to address this danger.

## 5. Conclusions

Subsistence farmers mostly had a formal education which helped those adopting recent storage innovations like using more metal tanks than traditional silos for storing their maize. Most of them had a farm size of 1 hectare and predominantly cultivated yellow maize. Metal tanks tended to be the most preferred storage facility for storing maize, even though this method is likely to favour pest infestation, especially of the weevils. Several weevil species are known to infest stored maize. Nonetheless, only one weevil species was reported, *S. zeamais*, and a moth, *S. cerealella*. There is a possibility that other stored maize pests occur in this region but were not detected during the current study. The study found that insect pest control tactics were dominated by chemical pesticides, namely aluminium phosphide. The concern for human health-related issues emanating from the use of this pesticide in preserving maize necessitates an effort to find alternative methods that are safe to supplement this current method. The current study also recommends training workshops to educate farmers on how to apply the recommended dosages and intervals between applications as per the guidelines. Further, farmers should be advised to rinse treated stored maize properly before consumption.

## Figures and Tables

**Table 1:** Socio-demographic information of small-scale farming in KSD municipality.

Variables		Frequency	Percentage (%)
Gender	Male	55	71.43
	Female	22	28.57
Age (years)	18–35	4	5.19
	36–55	25	32.47
	56+	48	62.34
Education level	None	12	15.58
	Primary	21	27.27
	Secondary	38	49.35
	Tertiary	6	7.79
Farming experience (years)	1	1	1.30
	2–5	11	14.29
	6–9	6	7.79
	10+	59	76.62

**Table 2:** Maize storage, storage form, and pest control practices.

Variables		Frequency	Percentage (%)
	Metal tanks	63	81.82
	Metal tanks and sacks in residential houses	8	10.39
	Sacks in residential houses	6	7.79
Storage form	Shelled maize	75	97.40
	Unshelled maize	2	2.60
Maize weevils infestation	Yes	69	89.61
	No	8	10.39
Other pests apart from maize weevils	Grain moths	57	74.03
Control practices	Use of chemical (Aluminium Phosphide)	65	84.42
	Use of chemical (Aluminium Phosphide) and other control practices (dry pepper, camphor, and wood ash)	10	12.99
	Doing nothing	1	1.30
	Removing affected grains	1	1.30

## Data Availability

The data presented in this study are available in the tables and figures of the current manuscript.
